# Optimizing methods and dodging pitfalls in microbiome research

**DOI:** 10.1186/s40168-017-0267-5

**Published:** 2017-05-05

**Authors:** Dorothy Kim, Casey E. Hofstaedter, Chunyu Zhao, Lisa Mattei, Ceylan Tanes, Erik Clarke, Abigail Lauder, Scott Sherrill-Mix, Christel Chehoud, Judith Kelsen, Máire Conrad, Ronald G. Collman, Robert Baldassano, Frederic D. Bushman, Kyle Bittinger

**Affiliations:** 1 Division of Gastroenterology, Hepatology, and Nutrition, The Children’s Hospital of Philadelphia, Philadelphia, Pennsylvania 19104 USA; 2 Department of Microbiology, University of Pennsylvania, Philadelphia, Pennsylvania 19104 USA; 3 Department of Medicine, Perelman School of Medicine, University of Pennsylvania, Philadelphia, Pennsylvania 19104 USA

**Keywords:** Metagenomics, 16S rRNA gene, Shotgun metagenomics, Environmental contamination, Methods, Study design, Best practices

## Abstract

Research on the human microbiome has yielded numerous insights into health and disease, but also has resulted in a wealth of experimental artifacts. Here, we present suggestions for optimizing experimental design and avoiding known pitfalls, organized in the typical order in which studies are carried out. We first review best practices in experimental design and introduce common confounders such as age, diet, antibiotic use, pet ownership, longitudinal instability, and microbial sharing during cohousing in animal studies. Typically, samples will need to be stored, so we provide data on best practices for several sample types. We then discuss design and analysis of positive and negative controls, which should always be run with experimental samples. We introduce a convenient set of non-biological DNA sequences that can be useful as positive controls for high-volume analysis. Careful analysis of negative and positive controls is particularly important in studies of samples with low microbial biomass, where contamination can comprise most or all of a sample. Lastly, we summarize approaches to enhancing experimental robustness by careful control of multiple comparisons and to comparing discovery and validation cohorts. We hope the experimental tactics summarized here will help researchers in this exciting field advance their studies efficiently while avoiding errors.

**Electronic supplementary material:**

The online version of this article (doi:10.1186/s40168-017-0267-5) contains supplementary material, which is available to authorized users.

## Background

Studies of microbial communities—the microbiome—have become quite popular in recent years. These studies are powered by the new DNA sequencing technologies which allow acquisition of over one trillion bases of sequence information in a single instrument run. Using these methods, sequence profiles of microbial communities from different sources can be obtained and compared to elucidate the associated patterns in the microbiota. For example, human samples from a disease state can be compared to samples from healthy controls, allowing for quantification of differences [1–8]. In these studies, DNA is first purified from the samples. DNA sequencing is then used to characterize the associated taxa, querying either a marker gene (16S for bacteria, 18S for eukaryotes, and ITS for fungi) or all DNAs in a mixture (shotgun metagenomics sequencing). In at least some situations, the nature of these microbial communities matters a lot—fecal microbial transplantation radically resets gut community structure and cures relapsing *Clostridium difficile* infection in up to 90% of cases [9, 10].

Carrying out definitive experiments on the microbiota requires great care, as in any field of research. All analytical methods have biases that must be taken into account in experimental execution and interpretation. For example, for analysis of 16S rRNA gene segments, the choice of gene region studied influences the types of bacteria queried [11–16]. Another example, emphasized here, involves low microbial biomass samples. If there is very little microbial DNA in a specimen, the library preparation and sequencing methods will often return sequences that are derived primarily from contamination [17–24]. Contaminating sequences can originate in reagents, dust, crossover between samples, or other sources. Without appropriate precautions and controls, these false calls can be difficult to distinguish from authentic microbiota. Other challenges mentioned below include changes associated with sample storage, microbial sharing among animals during cohousing, and authentic longitudinal microbial instability in the body site of a host animal.

The goal of this article is to catalog major challenges in microbiome research and to outline approaches to address them. Many of these points have come up in the projects of the PennCHOP Microbiome Program, with which the authors of this article are associated. This review is intended to help our collaborators and other microbiome researchers wrestling with these issues. We will focus primarily on laboratory work important for microbiome analysis and touch on computational and statistical methods only briefly. Most examples will be from 16S rRNA marker gene sequencing, but examples from ITS marker gene sequencing for fungi and shotgun metagenomics are also discussed. Several good articles have also addressed these issues and are recommended as additional reading [25–29]. Reviews of methods for bioinformatics analysis of microbiome specimens include [28, 30–33]. We focus here on studies of the vertebrate microbiome and break out points that are specific to studies of humans and model organisms. We present sections in an order that matches the progression of performing an experiment—the paper begins with study design, continues with sample collection and processing, and concludes with analysis.

## Planning a microbiome experiment

It is essential to plan carefully to ensure that the experiment carried out will answer the question posed. Plan the statistical analysis for your study at the start. If possible, carry out a power analysis. Several approaches tailored to microbiome research have been reported [34, 35].

### Consider the influence of factors such as antibiotic use, age, sex, diet, geography, and pet ownership

The human microbiome is sensitive to its environment, which can considerably confound associating any particular condition or intervention with a change in microbiota composition. Drug use, diet, age, geography, pet ownership, and sex have all been reported to influence function and composition [36–39]. In 2008, Relman and colleagues documented effects of antibiotic treatment on the gut microbiome, and many subsequent studies have also reported effects [5, 40–42]. It has further been suggested that additional prescription drugs can affect microbiome analyses [43, 44]. For example, Imhann et al. have suggested that decreasing the acidity of the stomach with proton pump inhibitors allows upper gastrointestinal microbes to move down into the gut more readily [45], altering the composition of the lower gastrointestinal microbiota and increasing the risk of *C. difficile* infections.

Diet also influences the microbiota [5, 46–56]. Microbial community structure and gene expression are reported to change on short-time scales in response to extreme short-term alterations in diet [57]. Long-term dietary patterns have been linked to gut microbiomes dominated by certain genera—diets high in protein and animal fat are associated with high *Bacteroides*, whereas diets high in carbohydrates are associated with high *Prevotella* [55].

The human microbiome evolves from birth until death. Typically, the gut microbiota adopts a stable anaerobic pattern around age 3 years but varies in early life [58–60]. The microbiome also changes in old age, with institutionalized elderly commonly developing high levels of *Proteobacteria* [61]. Thus, it is critical to use age-matched controls for microbiota comparisons.

Sex can also affect microbiome studies. The gut microbiome serves as a virtual endocrine organ due to the metabolites and neurotransmitters it produces [62]. For example, early microbial exposure has increased testosterone levels in male mice, leading to a protective effect against type 1 diabetes [63]. When the microbiota from these protected male mice was transplanted into younger female mice, the same protection against type 1 diabetes was seen [63]. A study of an anti-psychotic drug on weight and gut microbiota in male and female rats reported that drug treatment induced significant weight gain in female rats only [64]. Microbial circadian rhythms in mice were reported to differ between sexes [65]. Sex differences in microbiota have also been reported in macaques [39, 66].

Remarkably, even sexual preference among men has been linked to gut microbiome differences [67], which may be a confounding factor in studies of gut microbiome and HIV infection where controls were not matched by sexual preference.

Other studies have investigated whether pets influence the human microbiome and vice versa [68]. One group showed that cohabiting adults shared more similar skin microbiota if they owned a dog [69].

How each of these factors will influence any given microbiome study is dependent on the question asked and the strengths of differences between study groups. In general, it is important to enumerate possible confounders during experimental design, quantify each, and then treat them each as independent variables in downstream statistical analyses.

### Longitudinal instability

During experimental design, it is important to consider the longitudinal stability of the microbiota to be studied. The healthy human adult gut is known to be largely stable in microbial composition over time [70–72], and a perturbation in such stability—dysbiosis—has been associated with diseases such as inflammatory bowel disease [1, 5, 73]. However, the microbiome of other sites, like the human vagina, can vary on short-time scales without necessarily indicating dysbiosis [74–78]. Even the gut microbiome has been reported to display circadian behavior on a 24-h cycle [65, 79, 80]. Thus, for studies of a new sample type, it is essential to understand longitudinal variation in order to acquire samples that address the question posed.

Different batches of DNA extraction kit reagents can be a significant source of variation for longitudinal studies [23, 81]. It is wise to purchase all the extraction kits needed at the start of the study, or store samples and extract all at the same time, to minimize the effects of this variable.

### Cage effects in animal experiments

Cage effects can derail microbiome studies in mice and may be important for other laboratory animals as well. Mice housed in the same cage come to share similar gut microbiota due to mixing by coprophagia [82]. For perspective, in a recent study, mouse strain was found to account for 19% of the variation in gut microbiota, whereas cage effects contributed to 31% [83].

To account for cage effects, an investigator must set up multiple cages for each study group and treat the cage as a variable in the final statistical analyses. One can then determine whether microbial communities differ between groups given the measured effect of the cage variable. To keep costs down, it is fine to house two to three mice per cage [84–86].

**Fig. 1:**
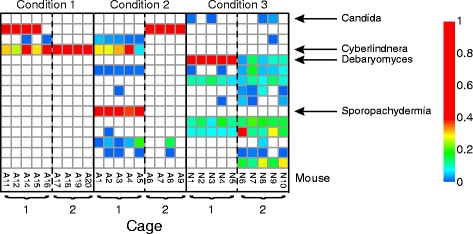
Example of cage effects dominating a mouse study of fungal communities. Fungal lineages in the murine gut were inferred from ITS rRNA gene sequencing of pellets [87]. The heat maps summarize taxonomic assignments derived from the sequence data. The color scale to the *right* indicates the proportions of each lineage; *white* indicates not detected. Caging dominated over treatment in this study. The three conditions studied were continuous exposure to antibiotics (*Condition 1*), short-term exposure to antibiotics (*Condition 2*), and no exposure to antibiotics (*Condition 3*). For details see [87]

As an example, consider the longitudinal study of fungal populations during an antibiotic intervention in mice in Dollive et al. [87]. In this work, antibiotic treatment was associated with increased fungal colonization in the treated groups (Fig. 1). The fungi detected were mostly consistent within each cage, but varied from cage to cage within each treatment group and also in the untreated controls. The types of fungi detected changed longitudinally, but nevertheless were consistent within cages. This highlights how potent cage effects can be, and emphasizes the importance of analyzing multiple cages per study group.

## Considerations during sample collection and processing

### Sample storage conditions

The most important considerations for storing microbiome samples are to reduce changes in the original microbiota from sample collection to processing and to keep storage conditions consistent for all samples in a study. Sample storage conditions are not always consistent between labs due to downstream applications and resource limitations. In 2010, Wu et al. compared human fecal samples that were immediately frozen at −80 °C, stored on ice for 24 h, or stored on ice for 48 h before DNA extraction and analysis. Differences due to storage method were not significant compared to differences between human individuals [88].

Due to an increased number of studies collecting samples from remote locations, several groups have assessed the efficacy of preservation methods that may be used when laboratory freezers are not readily available. In 2016, Song et al. tested the effects of different preservatives and temperature fluctuations on feces to mimic microbiome sampling in the field. If fecal samples cannot be frozen, store the samples in 95% ethanol, on FTA cards, or use the OMNIgene Gut kit [89]. These conditions are optimized for sample collection in the field; however, they may not be applicable to all studies depending on study goals and available resources. Other groups have also published similar sample storage studies [90–97].

**Fig. 2:**
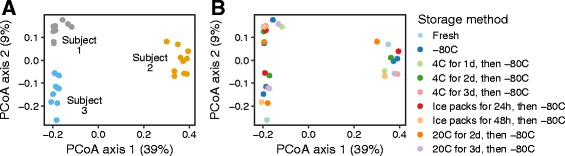
Effects of sample storage methods on community structure inferred for oral swabs. Oral swab samples were acquired from three human individuals and DNA extracted. DNAs were amplified using 16S rRNA gene primers binding to the V1-V2 region then sequenced using the Illumina platform using our standard procedures [88]. Unweighted Unifrac (C [129].) was used to generate distances between all pairs of samples then results were displayed using Principal Coordinate Analysis (PCoA). **a** Samples from each of the three subjects are color coded (*red*, *blue*, and *green*). **b** Nine storage conditions were compared, indicated by the different *colors*. The key to storage conditions is at the *right*

We recently performed a study on the storage of oral swab samples and found conditions to be relatively forgiving (Fig. 2). In this study, we collected cheek swab samples from three healthy subjects and stored them in a variety of conditions (frozen at −20 °C, refrigerated at 4 °C, or stored at an ambient temperature of 20 °C for 0, 24, 48, 72, or 96 h) before freezing at −80 °C (details and additional analysis are presented in Additional file 1). Figure 2 shows a principal coordinates analysis of unweighted UniFrac distance between the samples. The subject identifier (Fig. 2a) accounted for almost half the total variation in UniFrac distances (*R* ^2^ = 0.47, *P* < 0.001). The storage conditions did not represent a significant effect (Fig. 2b)—we estimated the relative effect size at less than half the effect of inter-subject variability (*R* ^2^ = 0.17, *P* = 0.2). The UniFrac results were recapitulated in our analysis of taxon abundances, where the effect of subject far exceeded any potential storage effects. This analysis provided evidence that over a period of 3 days, storage conditions of cheek swabs did not substantially influence the measured oral microbiome composition for these subjects. Another group recently investigated the effect of collection method, storage condition, and storage medium on taxonomic relative abundance in saliva and dental plaque, and found saliva samples stored in OMNIgene medium to be relatively consistent after a week at room temperature [98].

Optimal storage conditions have also been investigated for other sample types. Lauber et al. tested the effect of both temperature and length of storage on relative taxon abundance of bacterial communities in soil, human skin, and human fecal samples. The overall composition of bacterial communities and the relative abundance of most major bacterial taxa did not change with different storage conditions studied (*P* > 0.1 for all sample types) [99]. Replicate samples for both skin and feces clustered by host rather than by temperature or length of storage. However, Lauber et al. mentioned that one fecal sample replicate kept at room temperature was excluded from analysis due to visible fungal growth before DNA was extracted. Though convenience can be prioritized when handling samples over a short period of time (e.g., shipping samples on cold-packs for a 48 hour period before putting them in the freezer), we do recommend freezing samples promptly after collection or using alternative preservative methods if freezers are unavailable [89].

### Low microbial biomass samples—managing environmental contamination

Handling and analyzing samples with low microbial biomass can be challenging. Reagent and laboratory contamination comprise a larger proportion of the total microbial load in these samples compared to samples with rich microbial communities (e.g., healthy human feces). The low absolute amount of starting material can be overpowered by trace amounts of DNA from reagents or laboratory instruments used for sample processing, so that some or all of the microbial reads can be derived from environmental sources. Accounting for potential contaminants is especially important when studying the microbiome of body sites with low levels of bacteria, such as the human lung and skin, or sites that may not normally harbor any microbes at all, such as various healthy tissues [17, 19, 22].

Problems with contamination were well recognized even before the era of deep sequencing [100–102]. More recently, several groups have reported on the presence of bacteria in DNA extraction kits—the “kitome”—as well as other reagents used during sample processing [20, 23, 24, 103]. Salter et al. demonstrated that serial dilutions of a bacterial culture produced more contaminating 16S sequence reads and fewer “real” reads with each subsequent dilution, until contamination accounted for the majority of total sequences [23]. This pattern occurred at three different institutes that participated in this study, indicating a widespread issue [23]. Salter and colleagues also investigated effects of the number of PCR cycles for amplification. For low biomass samples, 20 cycles was too low, but 40 cycles recovered both contaminating and authentic low level sequences [23]. Later, Kennedy and colleagues reported that starting template concentration was the major factor behind variability in sequencing results [104]. Even in metagenomic samples prepared without a targeted PCR amplification step, similar contamination patterns were observed for samples containing low amounts of microbial DNA [23].

The kitome varies between kits, and can even vary between different lots of the same kit [20, 23]. Thus, it is best to process all samples in a project side by side using the same batches of reagents. It is crucial to record the kit used to process each sample, and which batch of each kit was used. If multiple kits were used, treat kit batch as a factor in the statistical analysis.

In our lab, we have investigated different DNA extraction methods in order to minimize the presence of the kitome. While the MO BIO PowerSoil DNA Isolation Kit (MO BIO Laboratories, Carlsbad, CA, USA) provides high yields and has been used widely in microbiome work, including the Human Microbiome Project [105], the kit was designed to isolate DNA from soil, stool, and environmental samples which are high in microbial DNA. The MO BIO kit was not manufactured with the intention of minimizing background contamination. *C. difficile* and *Streptophyta*, for example, have both been identified as possible reagent contaminants in this kit [22]. For low microbial biomass samples, we instead recommend using DNA isolation kits designed to minimize kit contamination (e.g., the QIAamp UCP (UltraClean production) Pathogen Mini Kit (QIAGEN)). Remember: it is important to choose one kit type for all of the samples in a microbiome study. Thus, if a project contains both low and high microbial biomass samples, please commit to one kit type for all samples in order to avoid kitome variation.

On the analytical side, several methods have been developed for filtering suspected contaminating taxa. In a study of the human oral and lung microbiome, Bittinger et al*.* introduced a method to determine the probability that fungal taxa arose from contamination sources [18], making use of the total fungal DNA concentration, as approximated by post-PCR assays of DNA concentration using PicoGreen. The PicoGreen assay is usually included in the sequencing protocol as a standard step, so the data is available with no extra effort. Similarly, Lazarevic et al. presented a method that incorporates measurements of total DNA concentration by qPCR, a more accurate but more resource-intensive approach [106]. Jervis-Bardy and colleagues showed that contaminating taxa tend to show a strong decrease in relative abundance as total DNA concentration increases and used this as the basis of another method to remove contaminant taxa [21]. Individual contamination sources can be modeled using SourceTracker, which employs a Bayesian approach to estimate the relative fraction of sequence reads arising from each source [107].

**Fig. 3:**
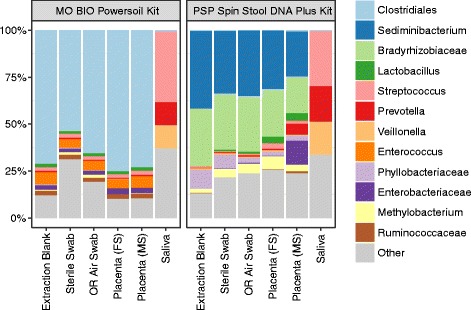
Wrestling with kit contamination—similar bacterial composition in placental samples and negative controls. Relative abundances of bacterial lineages were inferred from 16S V1-V2 rRNA marker gene sequence information [22]. Samples studied included negative controls, fetal side (FS) placental swabs, maternal side (MS) placental swabs, saliva, and vaginal swabs. Replicates of each sample were extracted using two different kits—the kit type is indicated above each panel. Operating room (OR) air swabs are swabs that were waved in the air at the time of sample collection to be used as negative controls. Saliva samples, which are high in microbial biomass, showed similar compositions for each of the two extractions; placental samples resemble the kit-specific negative controls

Studies investigating a potential placenta microbiome provide a case study of the difficulties of working with low biomass samples (Fig. 3). Several groups have reported that there may be a unique, low-abundance microbiome in healthy human placenta [46, 108–110], but reporting of negative controls in these studies has been incomplete.

However, a series of independent control studies showed no significant difference in taxonomic abundance between placenta samples and contamination controls [22]. Lauder and colleagues extracted DNA from placenta from six human subjects and worked them up alongside several types of blank swabs and empty extraction wells containing reagents only. DNA was extracted from samples using two different purification kits in order to characterize the contribution of the kitome. Real-time qPCR was performed to quantify total 16S rRNA gene copies in placental samples, controls, and saliva samples (from the same subjects) which were also purified using both DNA extraction kits. Placental samples and controls showed copy numbers that were low and indistinguishable from negative controls regardless of the kit used, whereas oral samples showed high signals several logs above background. Characterization of bacterial lineages by 16S rRNA gene sequencing showed that oral samples harbored distinct 16S profiles characteristic of the well-studied oral microbiota, and results were consistent between kits. However, placental and control samples looked similar to each other, but the pattern seen tracked with the DNA extraction kit used rather than with the sample type (Fig. 3). Several of the shared lineages found in placental and control samples were known contaminants of DNA extraction kits. The inference was that the kitome provided the predominant microbial signature in placental samples [22]. It remains to be seen whether future studies can show a clear distinction between placental samples and negative controls.

### Negative control samples

**Fig. 4:**
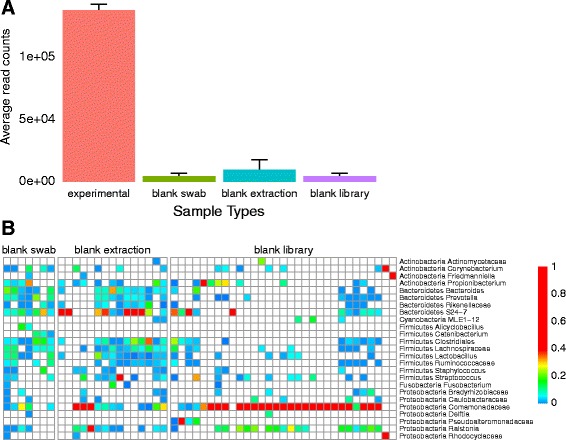
Analysis of three negative control sample types reveals contaminating taxa. Data for negative controls was acquired using 16S V1-V2 rRNA marker gene sequencing analyzed on the Illumina MiSeq platform. Data from 11 experiments were pooled. **a** Comparison of average read counts. Experimental samples had an average read count of 137,243 and negative control samples had an average read count of 6613. **b** Heat map summary of bacterial lineages present in negative control samples. Different OTUs are present in DNA-extraction controls (“blank extraction” and “blank swab”) and library preparation controls (“library blank”) collected over multiple sequencing runs

It is essential to collect negative control samples to allow empirical assessment of the contamination background. We commonly include three types of negative control samples on each 16S rRNA marker gene sequencing run (Fig. 4). In “blank swab” samples, a sterile swab was opened from its package in the sequencing lab, and the full sequencing protocol was applied to the swab. In “blank extraction” samples, DNA extraction and all subsequent steps were carried out with no additional input material. In “blank library” samples, the extraction protocol was not applied; DNA-free water (UltraClean PCR Water, MO BIO Laboratories, Carlsbad, CA, USA) was used as input to the post-extraction steps of the protocol, starting with library generation, to characterize contamination in downstream steps.

If microbial biomass is low, additional negative control samples can be included to measure contaminating DNA introduced during sample collection. As an example, in studies of the lung microbiome using bronchoalveolar lavage, an excellent negative control can be generated by washing the bronchoscope with a sample of the lavage saline prior to carrying out the bronchoscopy [19].

In our recent work, the average number of DNA sequence reads for negative control samples was typically five logs lower than the average for experimental samples derived from high biomass sites such as feces (Fig. 4a). The bacterial taxa appearing in negative control samples were among those previously reported as contamination in the literature, including *Comamonadaceae*, *Ralstonia*, and *Propionibacterium* (Fig. 4b).

### Positive control samples

Side by side sequencing of new samples with well-vetted positive controls is strongly recommended. Positive control samples allow verification that sample preparation and sequencing procedures are running smoothly. When samples are purified on multi-well plates, the consistent placement of samples in defined locations on plates allows any sample tracking mix-ups to be detected in the sequence output. Positive and negative controls will ideally be positioned asymmetrically on extraction plates, uniquely defining the plate orientation.

Many studies have used positive controls comprised of mixtures of cultured organisms (“mock communities”) [23, 96, 111] or known mixtures of free DNA (“mock DNA” samples) [88, 112, 113], both of which make useful controls. Analysis usually shows that sequencing results are reproducible within a method and lab environment, but biases can differ between methods and labs [23].

**Fig. 5:**
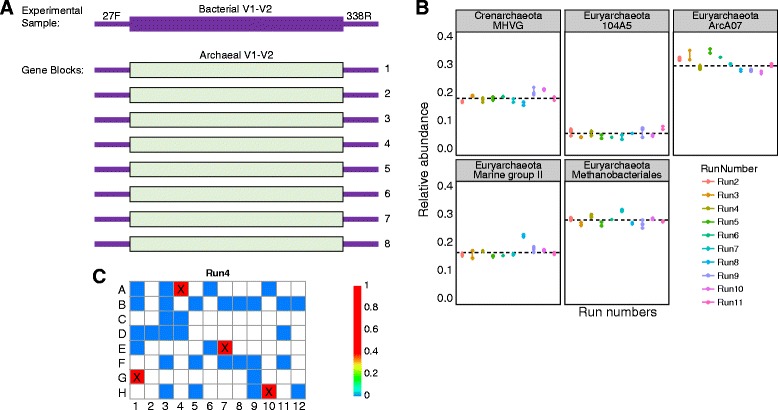
Synthetic non-biological 16S DNA as a positive control for 16S rRNA marker gene sequencing. **a** A diagram of the gene block design. At the *top* is a typical 16S rRNA gene amplicon, with primer binding sites for the widely used 27F and 338R primers. To generate recognizable sequences that would not be found authentically in samples, synthesized DNAs with the forward (27F) and reverse (338R) primer landing sites added to Archaeal DNA sequences, creating molecules not found in nature but readily analyzed using conventional pipelines. **b** Control sequence mixtures using the gene blocks show consistent relative abundances. Note that the eight gene blocks annotate as five archaeal taxa. **c** Heat map displaying the relative abundance of control gene blocks, where each square represents one well on a 96-well plate of a typical 16S rRNA marker gene sequencing project. Positive control wells where gene block was added and amplified alongside experimental samples are denoted with “x”

For a simple positive control, we designed and synthesized mock DNA samples as gene blocks (Fig. 5a, see Additional file 2 for DNA sequences). We selected DNA to synthesize using regions of the 16S rRNA gene in eight archaeal species which would not normally be detected in experimental data because the sequences at the amplification primer binding sites in the archaeal V1-V2 region do not match the bacterial V1-V2 primers used. In the engineered sequences, bacterial 16S V1-V2 primer binding sites were added synthetically to archaeal controls, allowing amplification. This has the advantage that the control sequences can be easily distinguished from experimental samples while still being processed through the same pipeline. A disadvantage of this strategy is that such controls are specific to a particular primer set and must be remade for each amplicon used. However, given the low cost of synthetic DNA, cost for a set of positive controls is modest (about $450). After sequencing archaeal gene block samples in 11 separate sequencing runs, we found that the relative abundances of the sequences were relatively consistent (Fig. 5b).

The gene block design provided an opportunity to test the level of cross-contamination between experimental samples during wet-lab library preparation (in 96-well plates) and sequence acquisition. Figure 5c shows representative results from one sequencing run. The abundance of control archaeal taxa did not increase with proximity to positive control samples on the 96-well plates (*P* = 0.6, linear regression analysis), suggesting that spill-over during preparation was not a prominent source of admixture between samples. However, low levels of these sequences could be detected in multiple dispersed samples (Fig. 5c, blue squares), potentially due to misreading of bar codes or hybridization of DNA molecules in adjacent clusters during Illumina sequencing [114]. A possible means of suppressing this would be to use bar codes on both ends of the amplicons and to require precise matches to both in the quality filtering [115].

The gene block scheme is a simple method for ensuring proper amplification of experimental samples, tracking sample mix-ups, and measuring sample cross-contamination during library preparation and sequencing. However, synthetic positive controls are not useful for benchmarking analytical and statistical methods. Analysis methods developed for real communities often do not perform as well on mock communities, and vice versa, due to the presence of naturally occurring sequence variation and low abundance taxa.

Many investigators use primers that simultaneously target the 16S region of both bacteria and archaea, for example, the 515fB/806rB primer set used by the Earth Microbiome Project [116, 117]. Here, there is no advantage to using archaeal sequences in the gene blocks because archaea might be observed in experimental samples. Nonetheless, investigators can build gene blocks using artificially altered DNA sequences that are different enough to be reliably distinguished from genomic sequence but similar enough to be compatible with the analysis pipeline. In Additional file 2, we present example gene block sets for the 515fB/806rB primer pair.

When artificial positive control samples are not suitable or cost effective, many of the benefits may be achieved by sequencing a small number of positive control samples collected from the field. We have used samples of pond water and saliva as indicators of consistency in sample preparation and sequencing, though ultimately found the mock DNA samples to be more convenient.

### Contamination in shotgun metagenomic data

Microbial DNA introduced by reagents can also be detected in shotgun metagenomic sequencing. As for amplicon sequencing, contamination is particularly apparent in samples with low microbial biomass. This is seen both for samples with generally low biomass (e.g., skin swab) and for samples dominated by non-microbial DNA (e.g., tissue biopsy).

**Fig. 6:**
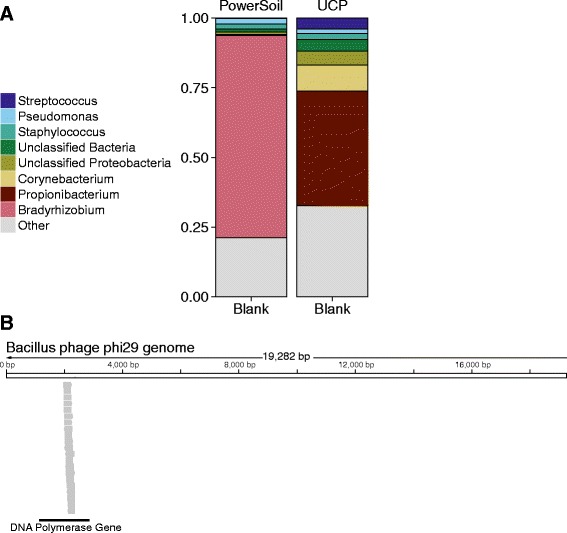
Contamination in shotgun metagenomic data. **a** Lineages observed in shotgun metagenomic sequencing of negative control samples using standard (DNeasy PowerSoil) and low-contaminant (QiaAmp UCP Pathogen) kits. **b** Detecting Bacillus phage phi29 polymerase reads in a blank sample. Twenty-one reads from a blank sample aligned to the DNA polymerase gene (1145 to 2863 bp) of Bacillus phage phi29. The protein was purchased as a reagent from a commercial supplier, suggestive of contamination of the protein with cloned DNA encoding the polymerase gene used in protein over-expression

For example, in our work to characterize the microbiota in sarcoidosis, we performed shotgun metagenomic sequencing on tissue DNA extracted using both standard (DNeasy PowerSoil, Qiagen, Valencia, CA, USA) and low-contaminant (QiaAmp UCP Pathogen, Qiagen, Valencia, CA, USA) kits (unpublished data). When sequencing negative control samples, we observed that the kit background differed between the two (Fig. 6a). Lineages found in both kits were also present in our low biomass tissue samples, likely derived from reagents. Lineages found in both samples and controls included *Propionibacterium* spp*.* and *Corynebacterium* spp., commonly associated with human skin, and *Bradyrhyzobium*, a common soil bacteria also identified as a contaminant by other groups [23, 118]. Of concern, this lineage has been proposed to be responsible for a colitis syndrome in patients undergoing umbilical-cord hematopoietic stem-cell transplantation [118, 119]—it will be key to strengthen the link to colitis with additional forms of data to rule out contamination as an explanation.

This indicates that while some reagent contamination is unavoidable, usage of low-contaminant kits reduces the total sequencing effort spent on contaminants. Furthermore, it highlights the importance of sequencing and analyzing extraction controls, because without them it is impossible to distinguish reagent contamination from true microbial signals.

An extreme example of contamination detection comes from virome analysis, where multiple displacement amplification is used to amplify specimens. The multiple displacement amplification method uses the phage phi29 DNA polymerase, a highly processive phage polymerase, to copy target DNA prior to library preparation. Shotgun metagenomic sequencing of a blank virome prep sample (unpublished data) returned hits on phage phi29, but upon inspection, these turned out to align exclusively to the polymerase gene (Fig. 6b). Evidently the amplification method was so sensitive that we recovered the gene used to produce a protein that we had purchased from a commercial supplier and used in our library preparation procedure.

## Considerations during analysis

This article is mostly concerned with optimal procedures for laboratory methods, but we do want to comment on three issues in analyzing and interpreting microbiome data.

### Handling of negative controls

It is essential to report compositions of negative control samples as for all other samples. Work up negative control samples through the full pipeline. Sequence negative control samples even if library yield is low or undetectable. Show the lineages present in stacked bar graphs or heat maps. Check negative control data into sequence archives when experimental samples are deposited. Do not just subtract lineages in negative controls and consider the problem solved. There is no reason to think that contaminating lineages are fully sampled without specific evidence, and there can be cases where environmental lineages are authentically present in samples and functionally important.

### Controlling multiple comparisons

High-throughput sequencing experiments commonly generate sequence reads attributed to hundreds of taxa. Researchers wishing to know which taxa are potentially associated with a difference in phenotype must make many comparisons, each time testing a null hypothesis of no difference in taxon abundance. In addition, studies will often involve multiple types of clinical data, allowing myriad comparisons over the microbiome data set. If the acceptable false positive rate for the test is set at a certain level (e.g., 5%), these repeated comparisons will raise the chances of getting a false positive higher than that level. To re-adjust the false positive rate back to the desired level, a multiple testing correction must be used.

This type of problem—controlling for multiple comparisons—is well covered by the statistical literature. A conservative approach is to ensure that none of the hypotheses are falsely rejected, within a specified probability, using the Bonferroni correction [120]. However, this method has been shown to be unacceptably conservative, leading to too many false negatives. A more popular approach is to control for a pre-specified rate of false discovery (i.e., false rejections of the null hypothesis). Benjamini and Hochberg presented a method to control for the false discovery rate in a series of independent tests [121], and this is the formulation used in microbiome analysis software such as QIIME [122] and Mothur [123]. Use of a multiple testing correction is strongly recommended whenever multiple comparisons are made.

### Discovery and validation cohorts

Moving beyond single experiments, researchers can provide better and more reliable evidence for a discovery by re-producing the results in an independent cohort of samples. The use of separate discovery and validation cohorts is standard in genome-wide association studies, which are also massively multivariate (e.g., [124].). Using this strategy in the microbiome context, the experiment is first conducted in the discovery cohort and taxa or gene types are selected using a particular testing procedure. The validation cohort is then analyzed to test only those results found to be significant in the discovery cohort. The total number of tests is thus drastically reduced in the validation cohort.

Several microbiome studies have used independent discovery and validation cohorts to select taxa of interest for a disease state. Sabino et al*.* identified three bacterial genera associated with primary sclerosing cholangitis in a discovery cohort and used their results to correctly classify 75% of subjects in an independent validation cohort [125]. Forslund et al. used separate cohorts to replicate their findings of taxa altered in metformin-treated subjects with type 2 diabetes mellitus [126]. In a series of papers, a composite index of bacterial taxon abundance in stool associated with inflammatory bowel disease (IBD) was developed in one group of subjects [73], and then found to distinguish IBD from healthy controls in an independent follow-up study [127]. Kelsen et al. applied the discovery-validation cohort design to determine differences in the subgingival microbiota between children with Crohn’s disease and healthy controls [128], and successfully demonstrated reproducible taxa. Additionally, they were able to distinguish taxa that were associated with antibiotic use from those associated only with the disease.

## Conclusions

Summarizing the considerations above, we can make several recommendations for the design and execution of microbiome studies.

- For analysis, multiple confounding factors need to be taken into account, including antibiotic use, age, sex, diet, geography, and pet ownership.
- In animal studies, cage effects can dominate over what may seem to be extreme interventions. Thus, it is critical to set up each condition to be studied in multiple cages, so that the caging variable can be isolated and accounted for.
- Although we recommend storing samples, especially fecal samples, at −80 °C immediately after collection for most accurate results, alternative storage methods for field studies also lead to results with relatively small deviations. For new sample types, it will be wise to test for changes during storage under study-specific storage conditions.
- In a cross-sectional study, it is essential to know whether the time point sampled will be representative. For example, the healthy adult gut microbiota does not change radically over short time scales, but that of the vagina sometimes does. Therefore, it is important to assess the relationship of possible longitudinal dynamics to the question posed.
- Be energetic in creating and analyzing negative controls—DNA extraction kits usually come with contaminants, and contamination may vary between suppliers and even between batches of the same kit.
- Use positive controls for each batch of samples. Mock communities are valuable for this, and the simple synthetic DNA controls presented here (Additional file 2) are also quite useful. Place controls asymmetrically in purification plates to verify proper sample tracking through the DNA purification and library preparation procedures.
- Low microbial biomass samples present many challenges. When starting a study that might involve low microbial biomass samples, it is essential to quantify the microbial load in the samples to understand the extent of the challenge. QPCR of total 16S rRNA gene copies can be used for this purpose, as can conventional plating assays if applicable. In an experiment that may involve low biomass samples, start with the null hypothesis that all sequence data reflects contamination only, and ask whether this idea can be rejected in a statistical analysis of the data.
- Be realistic about “data dredging,” that is, imposing a rigorous statistical method to control multiple comparisons.
- Lastly, if affordable, it greatly strengthens a study to assess effects in separate discovery and validation cohorts.

There is no question that the human microbiota are critical for health and disease—by attending to the above challenges, one can generate high quality data to drive new discoveries in this exciting field.

## Additional files

Additional file 1: Supplementary methods. (PDF 1926 kb)

Additional file 2: DNA sequences for gene block control samples. (XLSX 11 kb)

## Abbreviation

ITS: Internal transcribed spacer
