# Supplementary material for: Optimizing methods and dodging pitfalls in microbiome research
Source: Microbiome. 2017 May 5;5:52. doi: 10.1186/s40168-017-0267-5 (PMC5420141; doi:10.1186/s40168-017-0267-5)
Supplement: Supplementary file 1 — Supplementary methods. (PDF 1926 kb) [file 40168_2017_267_MOESM1_ESM.pdf]

Optimizing methods and dodging pitfalls in microbiome research: Additional File 1

Oral swab storage study

Cheek swab samples were obtained from three healthy volunteers and stored according to nine different methods, listed below.

	Day 1	Day 2	Day 3	Days 4-7	Day 8	Day 9	Day 10
Swab 1	4°C	4°C	4°C	-80°C	-80°C	-80°C	-80°C
Swab 2	RT	RT	RT	-80°C	-80°C	-80°C	-80°C
Swab 3	-80°C	-80°C	-80°C	-80°C	-80°C	-80°C	-80°C
Swab 4		4°C	4°C	-80°C	-80°C	-80°C	-80°C
Swab 5		Ice packs	Ice packs	-80°C	-80°C	-80°C	-80°C
Swab 6		Ice packs	-80°C	-80°C	-80°C	-80°C	-80°C
Swab 7			4°C	-80°C	-80°C	-80°C	-80°C
Swab 8			-80°C	-80°C	-80°C	-80°C	-80°C
Swab 9					RT	RT	-80°C
Swab 10							Fresh

Sample preparation and sequencing

DNA was extracted using the MO BIO PowerSoil kit. The V1-V2 variable regions of the bacterial 16S rRNA gene were amplified using barcoded primer sequences 27F (AGAGTTTGATCCTGGCTCAG) and 338R (TGCTGCCTCCCGTAGGAGT), as described previously[1]. Purified products were pooled in equal amounts and sequenced on the Illumina MiSeq. The paired-end sequencing protocol was used, yielding 250bp reads in the forward and reverse directions.

Bioinformatics processing

DNA sequence data was analyzed using QIIME version 1.9.1[2], followed by additional analysis in the R Language for Statistical Computing[3]. Read pairs were assembled to form a complete sequence for the V1-V2 variable region of the 16S rRNA gene, using a minimum overlap of 35bp and a maximum difference of 15%. The resultant sequences were quality filtered, using a minimum quality threshold of Q20. The sequences were clustered into operational taxonomic units (OTUs) using UCLUST[4], according to the *de novo* OTU clustering workflow in QIIME. Taxonomic assignments were performed against the Greengenes database[5], version 13_8, using the default method in QIIME 1.9.1. Representative sequences for each OTU were aligned with PyNAST[6], and a phylogenetic tree was estimated with FastTree[7]. Weighted and unweighted UniFrac distances were computed between each pair of samples[8].

Sequencing results

We collected 2.3 million total reads, with a median value of 59,000 reads per sample. Oral swab samples consisted of typical oral taxa, such as *Streptococcus*, *Veillonella*, *Rothia*, *Prevotella*, and *Haemophilus* (Fig. A1). Positive control samples from pond water contained a number *Proteobacteria*, *Firmicutes*, and *Chloroflexi* groups, which were rarely observed in the oral swab samples. Our blank extraction sample consisted predominantly of *Streptophyta*, which has been previously identified as a contaminant of extraction kits[9]. The community composition of positive and negative control samples was significantly different from that of oral swabs, when compared using unweighted UniFrac distance (Fig. A2, PERMANOVA test, R^2 = 0.26, P = 0.002). Results were similar with weighted UniFrac distance (R^2 = 0.51, P = 0.001).

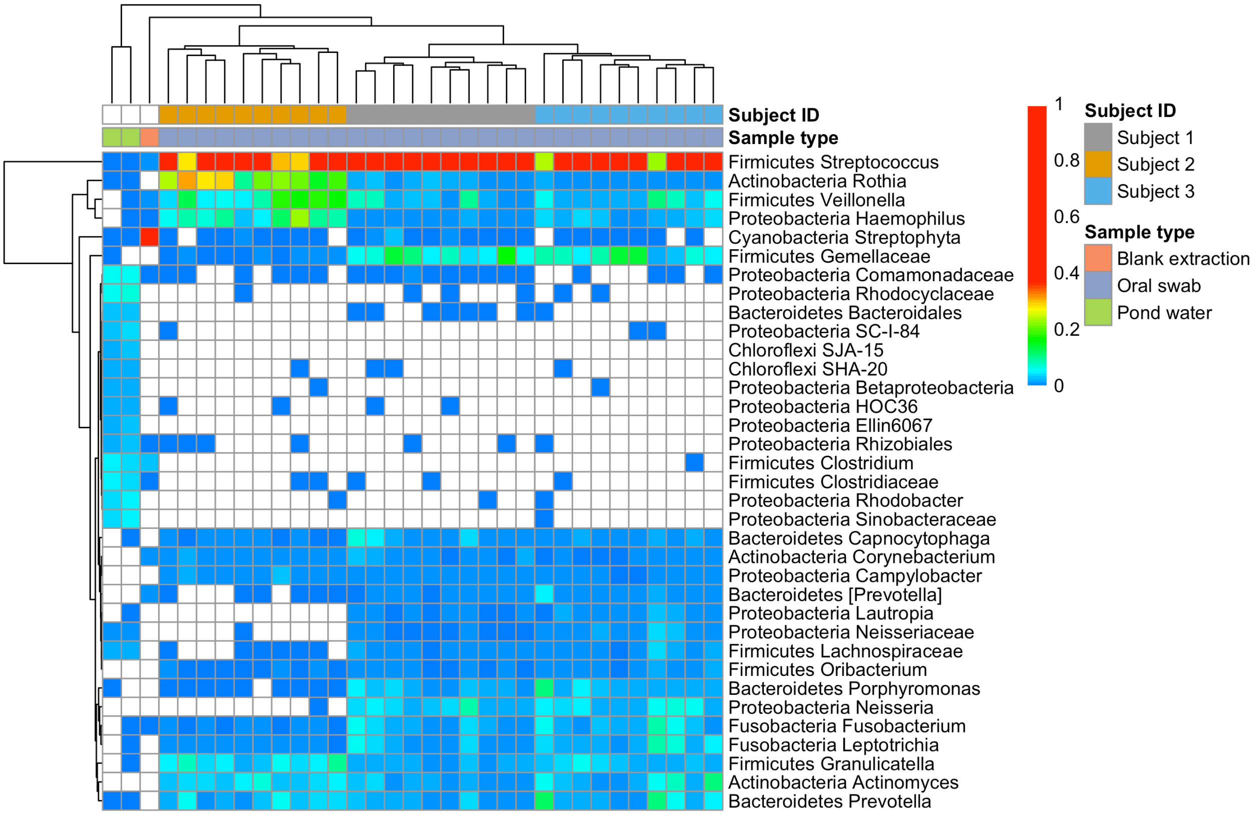

Figure A1. Heatmap of genus-level bacterial taxa proportions in cheek swab samples and control samples. Each column represents one sample. White squares represent taxa not detected in the sample.

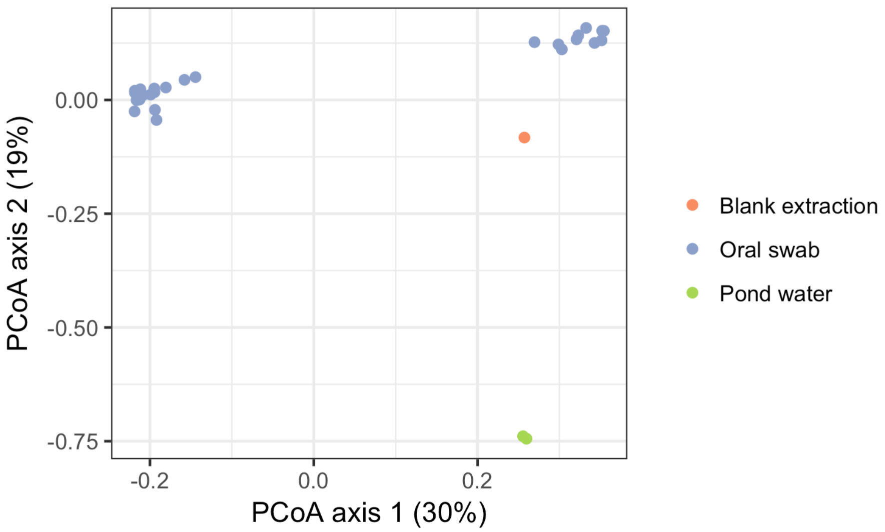

Figure A2: Principal coordinates analysis (PCoA) of unweighted UniFrac distance between oral swab samples and control samples.

<u>Analysis of storage methods</u>

The storage method used had no detectable effect on the within-sample diversity (richness, Fig. A3), when tested with a repeated-measures ANOVA ($P = 0.7$) or a linear mixed-effects model ($P = 0.6$). Conversely, the subject ID was statistically significant when included as a random effect ($P < 0.001$).

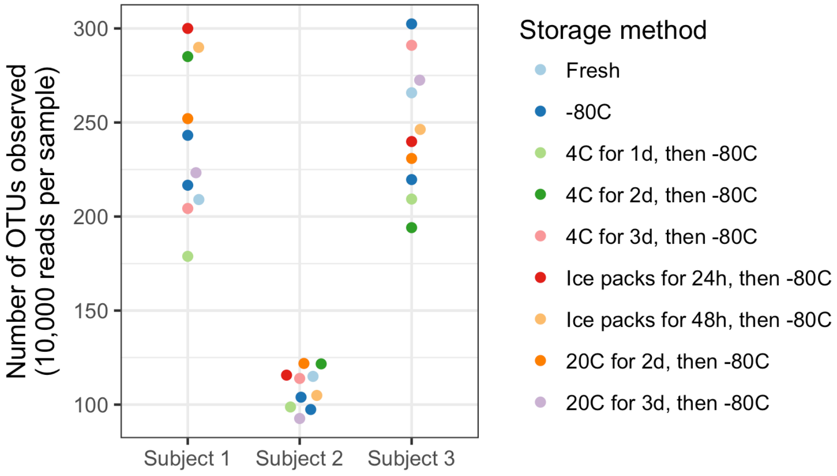

Figure A3: Bacterial richness oral swab samples, quantified by number of operational taxonomic units (OTUs) observed. To minimize bias due to unequal sample size, the expected value is shown for a sample size of 10,000 reads.

We compared the community composition of oral samples using weighted and unweighted UniFrac distance. Based on unweighted UniFrac distance, samples from the same subject appeared more similar than samples from different subjects (Fig. A4, PERMANOVA test, R^2 = 0.47, P = 0.001). Storage method did not have a statistically significant effect on unweighted UniFrac distance (Fig. A5, P = 0.2). Our results were similar using weighted UniFrac distance: we observed a significant effect of subject ID (Fig. A6, R^2 = 0.38, P = 0.04) but not of storage method (Fig. A7, P = 0.9).

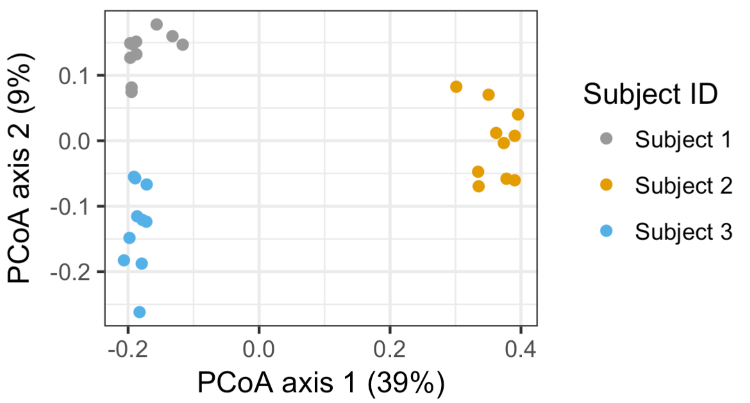

Figure A4: Principal coordinates analysis of unweighted UniFrac distance between oral swab samples, colored by subject.

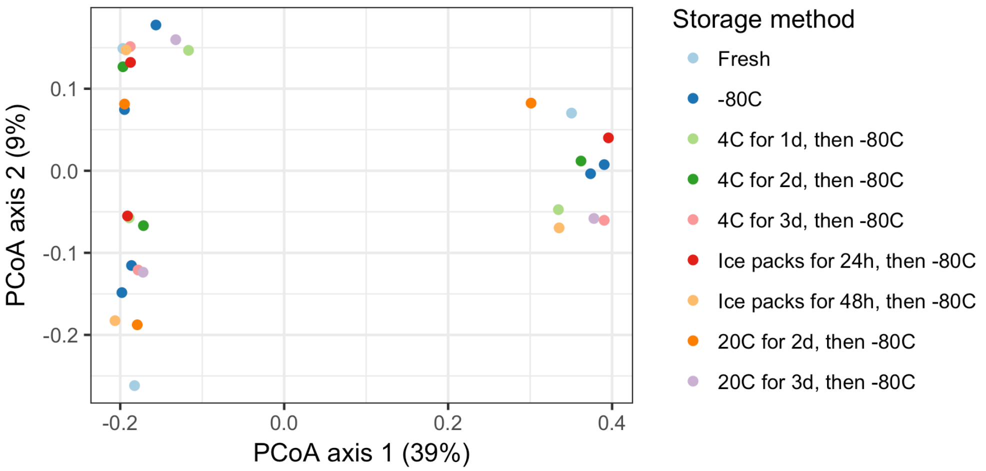

Figure A5: Principal coordinates analysis of unweighted UniFrac distance between oral swab samples, colored by storage method.

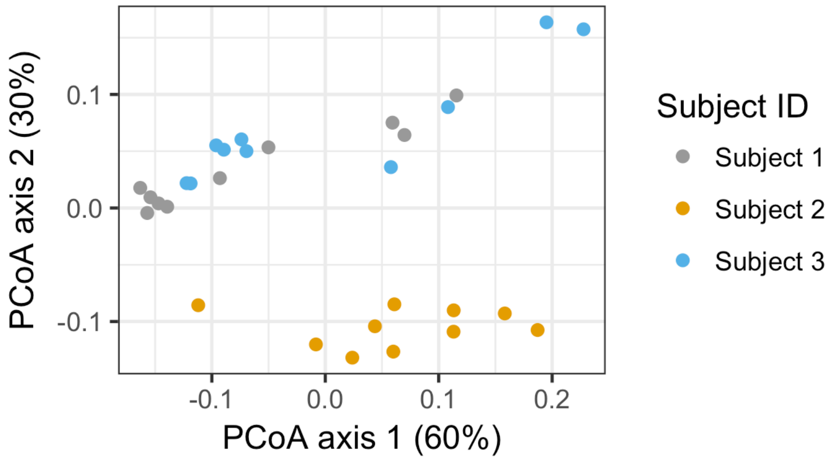

Figure A6: Principal coordinates analysis of weighted UniFrac distance between oral swab samples, colored by subject.

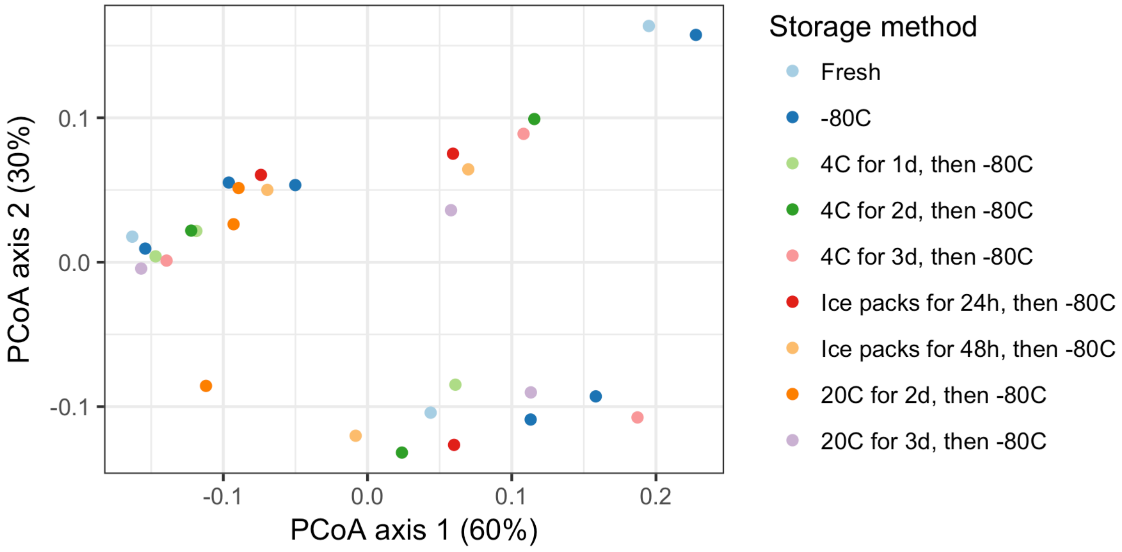

Figure A7: Principal coordinates analysis of weighted UniFrac distance between oral swab samples, colored by storage method.

To see if the method of storage was associated with alterations in specific bacterial taxa, we identified the top 12 most abundant bacterial genera in oral swab samples (Fig. A8). We tested the effect of storage condition using a linear mixed model on log-transformed proportion (Fig. A9), with subject ID included as a random effect. We observed no statistically significant effect for any storage method on any of the top 12 taxa. The minimum p-value observed was 0.06, before correction for multiple comparisons.

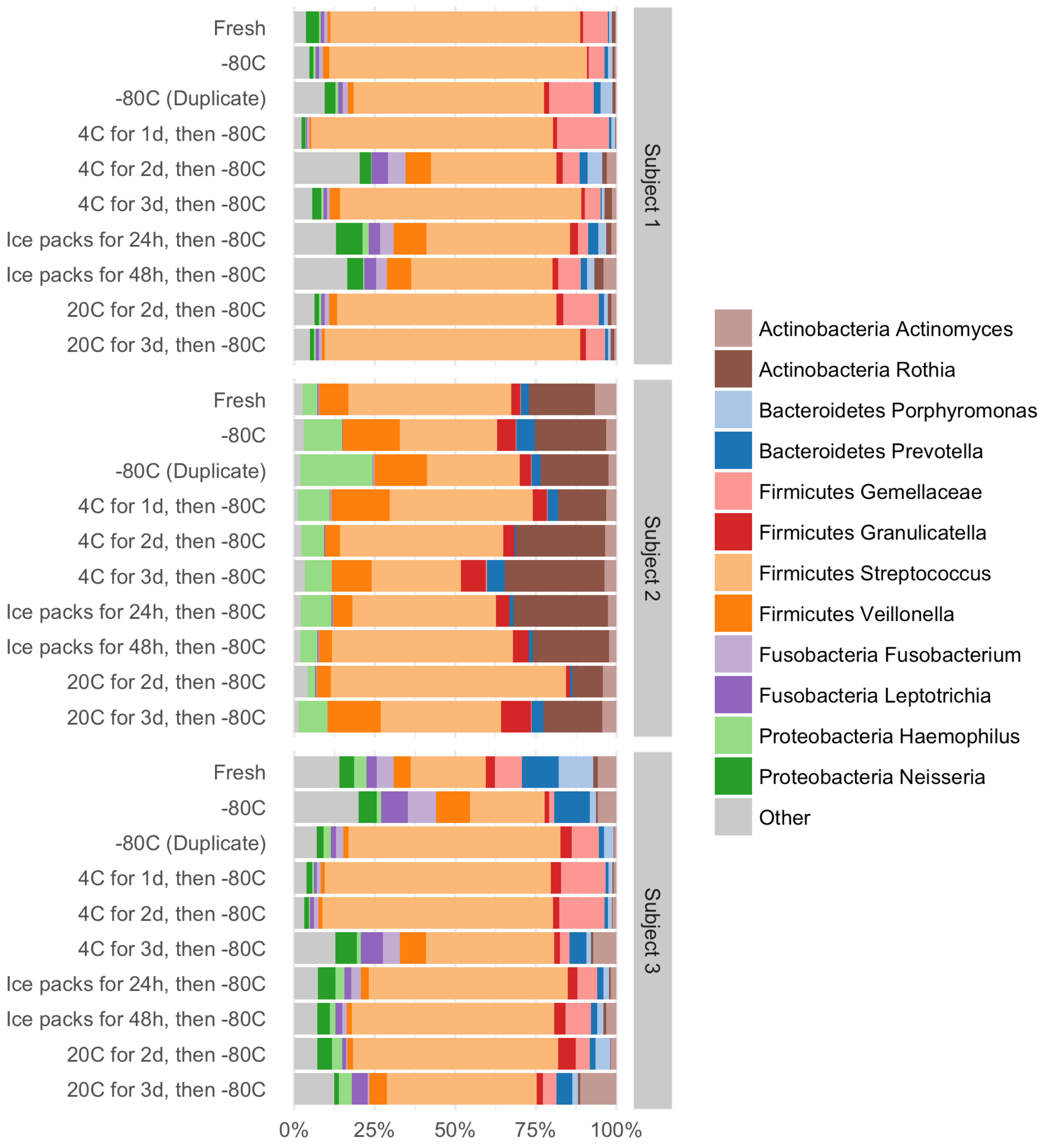

Figure A8: Proportions of the top 12 most abundant bacterial taxa observed in oral swab samples.

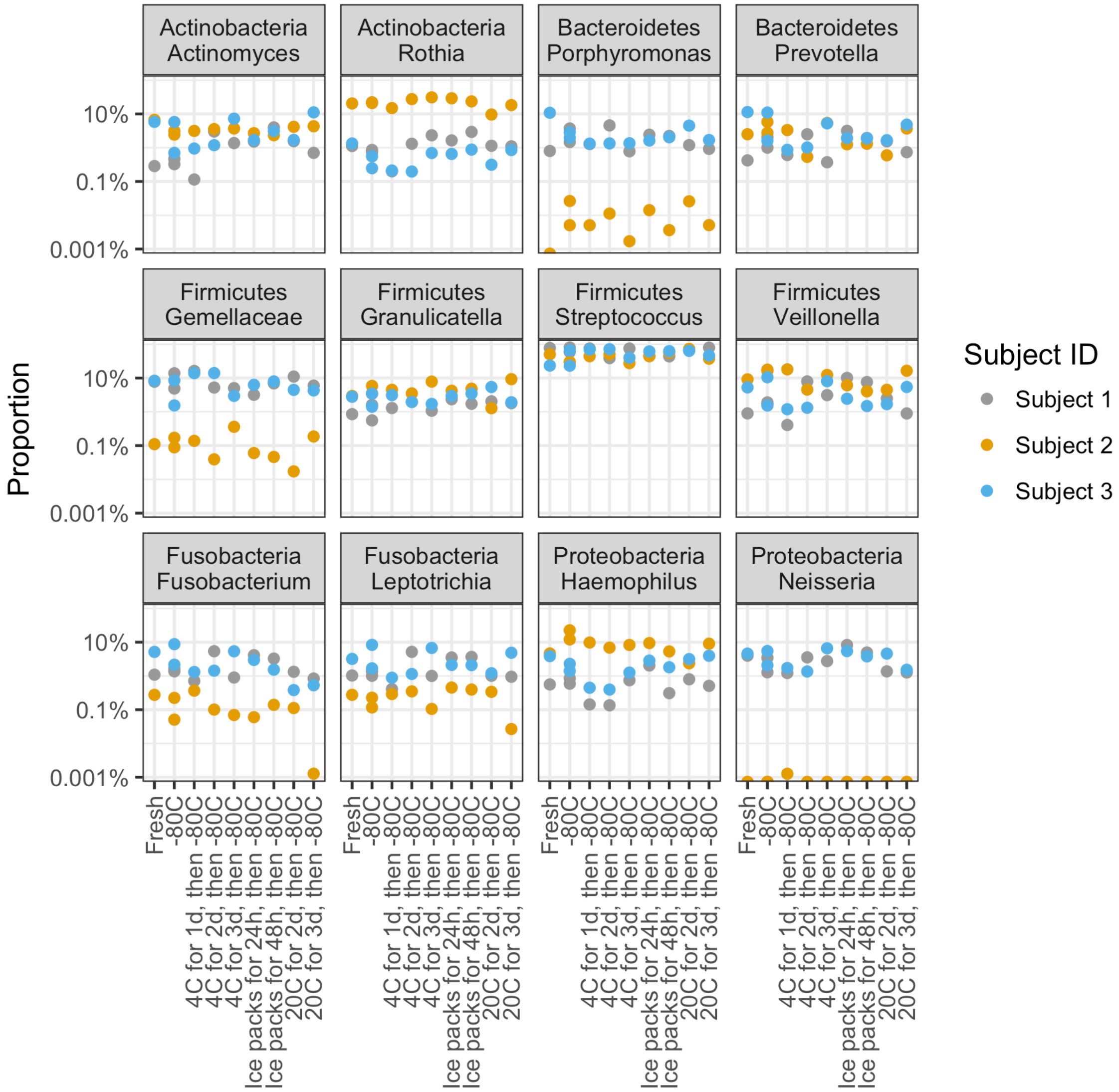

Figure A9: Bacterial taxon proportions shown across all storage conditions.

References

1. Wu, G. D., J. D. Lewis, C. Hoffmann, Y. Y. Chen, R. Knight, K. Bittinger, J. Hwang, J. Chen, R. Berkowsky, L. Nessel, H. Li, and F. D. Bushman. 2010. Sampling and pyrosequencing methods for characterizing bacterial communities in the human gut using 16S sequence tags. BMC Microbiol. 10: 206.
2. Caporaso JG, Kuczynski J, Stombaugh J, Bittinger K, Bushman FD, Costello EK, Fierer N, Gonzalez Pena A, Goodrich JK, Gordon JI, Huttley GA, Kelley ST, Knights D, Koenig JE, Ley RE, Lozupone CA, McDonald D, Muegge BD, Pirrung M, Reeder J, Sevinsky JR, Turnbaugh PJ, Walters WA, Widmann J, Yatsunenko T, Zaneveld J, Knight R. 2010. QIIME allows analysis of high-throughput community sequencing data. Nature Methods 7(5): 335-336.
3. R Core Team (2016). R: A language and environment for statistical computing. R Foundation for Statistical Computing, Vienna, Austria. https://www.R-project.org/.
4. Edgar RC. 2010. Search and clustering orders of magnitude faster than BLAST. Bioinformatics 26(19): 2460-2461.
5. McDonald D, Price MN, Goodrich J, Nawrocki EP, DeSantis TZ, Probst A, Andersen GL, Knight R, Hugenholtz P. 2012. An improved Greengenes taxonomy with explicit ranks for ecological and evolutionary analyses of bacteria and archaea. ISME J 6(3): 610–618.
6. Caporaso JG, Bittinger K, Bushman FD, DeSantis TZ, Andersen GL, Knight R. 2010. PyNAST: a flexible tool for aligning sequences to a template alignment. Bioinformatics 26:266-267.
7. Price MN, Dehal PS, Arkin AP. 2010. FastTree 2-Approximately Maximum-Likelihood Trees for Large Alignments. Plos One 5(3): e9490.
8. Lozupone C, Knight R. 2005. UniFrac: a new phylogenetic method for comparing microbial communities. Appl Environ Microbiol 71(12): 8228-8235.
9. Lauder AP, Roche AM, Sherrill-Mix S, Bailey A, Laughlin AL, Bittinger K, Bushman FD. 2016. Comparison of placenta samples with contamination controls does not provide evidence for a distinct placenta microbiota. *Microbiome, 4*(1): 29.
